# Urine biomarker score captures response to induction therapy with lupus nephritis

**DOI:** 10.1007/s00467-023-05888-z

**Published:** 2023-01-30

**Authors:** Ellen M. Cody, Scott E. Wenderfer, Kathleen E. Sullivan, Alfred H. J. Kim, Wesley Figg, Harneet Ghumman, Tingting Qiu, Bin Huang, Prasad Devarajan, Hermine I. Brunner

**Affiliations:** 1 Division of Nephrology, Hypertension and Pheresis, Department of Pediatrics, Washington University in St. Louis School of Medicine, 660 S. Euclid Ave, Campus, Box 8116, St. Louis, MO 63110 USA; 2 Department of Pediatrics, University of British Columbia, Vancouver, BC Canada; 3 Nephrology, B.C. Children’s Hospital, Vancouver, BC Canada; 4 Division of Allergy and Immunology, Children’s Hospital of Philadelphia, Philadelphia, PA USA; 5 Division of Rheumatology, Department of Medicine, Washington University School of Medicine, St Louis, MO USA; 6 Medical School, Baylor College of Medicine, Houston, TX USA; 7 Department of Rheumatology, Cincinnati Children’s Hospital Medical Center, Cincinnati, OH USA; 8 Division of Biostatistics and Epidemiology, Cincinnati Children’s Hospital Medical Center, Cincinnati, OH USA; 9 Division of Nephrology and Hypertension, Cincinnati Children’s Hospital Medical Center, Cincinnati, OH USA; 10 Department of Pediatrics, University of Cincinnati, Cincinnati, OH USA

**Keywords:** Lupus nephritis, Urine, Biomarker, Induction

## Abstract

**Background:**

The Renal Activity Index for Lupus (RAIL) consists of urine protein assessment of neutrophil gelatinase–associated lipocalin, kidney injury molecule-1, monocyte chemotactic protein 1, adiponectin, hemopexin, and ceruloplasmin, which non-invasively identifies lupus nephritis (LN). We aimed to delineate RAIL scores with inactive versus active LN and changes over time with response to LN induction therapy.

**Methods:**

There were 128 pediatric patients with systemic lupus erythematosus (SLE) and age-matched healthy controls recruited in a prospective case control study, with kidney biopsy confirmation of LN. Laboratory and clinical information was recorded and urine collected at diagnosis and end of induction and during maintenance therapy. Response to therapy was assessed by repeat kidney biopsy or laboratory parameters. Urine was assayed for RAIL biomarkers and the RAIL score calculated.

**Results:**

Pediatric RAIL (pRAIL) scores from 128 children and young adults with SLE (with/without LN: 70/38) including 25 during LN induction therapy, differentiated clinically active LN from inactive LN or without LN, and controls (all *p* < 0.0017). pRAIL scores significantly decreased with complete LN remission by 1.07 ± 1.7 (*p* = 0.03).

**Conclusions:**

The RAIL biomarkers differentiate LN patients based on activity of kidney disease, with decreases of ≥ 1 in pRAIL scores indicating complete response to induction therapy. Significantly lower RAIL scores in healthy controls and in SLE patients without known LN raise the possibility of subclinical kidney disease.

**Graphical abstract:**

**Figure:**
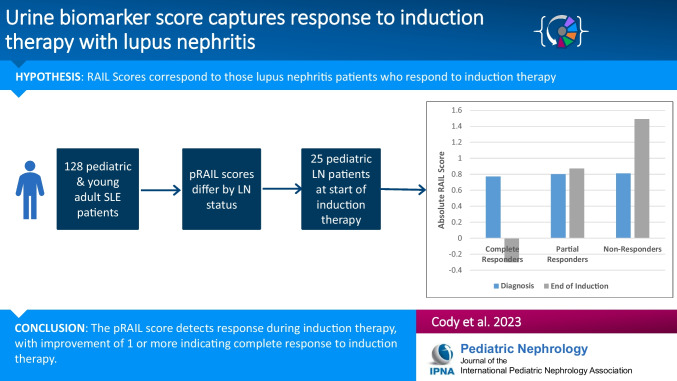
A higher resolution version of the Graphical abstract is available as Supplementary information

**Supplementary Information:**

The online version contains supplementary material available at 10.1007/s00467-023-05888-z.

## Introduction

Kidney involvement with systemic lupus erythematosus (SLE), or lupus nephritis (LN), continues to account for sizable morbidity, mortality, and kidney failure [1]. An estimated 20% of SLE cases begin in childhood onset (cSLE), and LN occurs in 32–37% of them, more frequently than in adult onset SLE [2–4]. Current clinical and laboratory measures failed to diagnose LN or capture LN activity reliably [1, 5, 6]; thus, invasive kidney biopsies are required for diagnosis, treatment, and prognosis [7–10]. A recent study suggests that about 30% of LN patients who are clinically classified as complete responders have residual kidney inflammation on repeat kidney biopsy upon completion of induction therapy [7, 8].

To provide accurate non-invasive means of monitoring LN disease activity and response to LN therapy, we discovered and validated a urine biomarker panel consisting of 6 urine proteins: neutrophil gelatinase–associated lipocalin (NGAL), monocyte chemoattractant protein-1 (MCP-1), kidney injury molecule-1 (KIM-1), ceruloplasmin, adiponectin, and hemopexin. Renal Activity Index for Lupus (RAIL) scores, with or without standardization of urine creatinine levels, are calculated from the urine concentrations of urine proteins based on patient age. There is a pediatric RAIL (pRAIL) algorithm and one for adults (aRAIL). A standardized pRAIL score of 0.39 or higher correctly identifies 92% of histologically proven, active LN in patients aged < 24 years [11, 12]. Likewise, the standardized adult RAIL score of − 0.97 or higher correctly identifies older patients with active LN with 86% accuracy [13]. We have also shown that the accuracy of pRAIL scores to identify active LN does not require urine creatinine adjustment, and that scores ≥ 2 are consistent with a NIH Activity Index (NIH-AI) value of 10 or higher, while pRAIL scores of 0 or less are consistent with NIH-AI values of 2 or less [14]. RAIL scores decrease (or improve) with immunosuppressive therapy in those with treatment response [11, 12].

We hypothesized that the improvement in RAIL score will detect those who respond to induction therapy versus those who do not. We evaluated RAIL scores based on the clinical activity of LN and compared RAIL scores to those in healthy controls. In this study, we aimed to delineate changes in RAIL scores during induction therapy of LN.

## Material and methods

### Patients

We performed a prospective case control study. With approval of local institutional review boards of the participating academic centers at Cincinnati Children’s Hospital (CCHMC), Children’s Hospital of Philadelphia, Washington University School of Medicine, and Baylor College of Medicine, clinical data and urine samples of patients with cSLE were included in this study. Study participants were pediatric patients diagnosed with SLE by the 1997 or the 2019 American College of Rheumatology (ACR) classification criteria [15, 16], irrespective of degree of SLE activity, presence/absence of LN. We excluded those with a diagnosis of other kidney diseases, or other autoimmune or inflammatory conditions besides SLE. Age-matched healthy controls were recruited and studied at CCHMC (IRB 2017–0585).

SLE patients were classified as having LN based on the result of a kidney biopsy [17]. Patients with LN contributed two or more visits within a 1-year period, while cross-sectional assessment occurred for all patients. Subgroup analysis included LN patients undergoing induction therapy, who had to be enrolled at time of LN diagnosis and followed for 6–9 months, with urine collected at the time of biopsy and at 6-month intervals until the end of induction therapy. We used REDCap (Research Electronic Data Capture, Vanderbilt University, Nashville, TN, USA) [18], a secure, web-based application designed to support data capture for research.

### Demographic, clinical, and laboratory data of patients

Besides demographics, information needed to score the SLE Disease Activity Index (SLEDAI) and the renal domain score of the SLEDAI (renal SLEDAI) was extracted [19]. This included results of urinalysis and measured urine protein to creatinine ratio (UPCR, in mg/mg) in spot urine at each visit [20]. The estimated glomerular filtration rates (eGFR) were calculated by modified Schwartz equation in the younger subjects and by CKD-EPI for patients above 18 [21, 22]. For patients with LN, we also recorded the results of kidney biopsies. Kidney histology was interpreted using the International Society of Nephrology/Renal Pathology Society (ISN/RPS) classification system, which included histologic activity and chronicity as measured by NIH-AI and NIH-CI [23–26].

### Biomarker measurements and RAIL scores

Urine collected was spun at 2200 × g for 10 min and stored at − 80 °C for no longer than 5 years. Human NGAL and human urine creatinine were measured on a Roche Cobas c 311 clinical chemistry analyzer using a commercially available assay (BioPorto, Denmark, Catalog KIT ST001RA for NGAL; Roche Diagnostics, Indianapolis, IN, Reference 03,263,991 190 for Creatinine). NGAL had a lower limit of detection of 9.8 ng/mL while creatinine had a lower limit of detection of 1.1 mg/dL. The remaining biomarkers were measured via ELISA, all done in duplicate. Human urinary KIM-1 (R&D Systems, Minneapolis, MN, DKM100) had a mean minimal detectable dose of 0.009 ng/mL. Human MCP-1 (R&D Systems, Minneapolis, MN, DCP00), diluted 1:1, had a mean minimal detectable dose of 1.7 pg/mL. Human adiponectin (R&D Systems, Minneapolis, MN, DRP300), diluted 1:5, had a mean minimal detectable dose of 0.246 ng/mL. Human ceruloplasmin (Assaypro LLC, St. Charles, MO, EC4201-1), diluted 1:50, had a mean minimal detectable dose of 0.085 ng/mL. Human hemopexin (Assaypro LLC, St. Charles, MO, EH2001-1), diluted 1:20, had a mean minimal detectable dose of 4.2 ng/mL. KIM-1 and MCP-1 used a four-parameter logistic curve to fit the standard curve. Adiponectin, ceruloplasmin, and hemopexin used a log/log curve to fit the standard curve. Analyte concentrations that were above or below the limits of detection were imputed by 50% of the level of lower limit of detection and 50% over the upper limit of detection, respectively.

### Definitions of active LN and course of LN

There is no consensus how to define active LN based on the NIH-AI scores; scores from 2 to 6 have been proposed previously [27–30]. For this analysis, we defined active LN as a NIH-AI score of ≥ 4 within 3 months of a study visit. Patients who had an NIH-AI score of < 4 were classified as inactive nephritis, regardless of ISN/RPS class. All LN patients required a kidney biopsy with urine collection at disease onset to be included in the study induction arm. In the setting of unavailable kidney biopsy within 3 months of a study visit, active LN was defined as a UPCR of ≥ 0.5 mg/mg *and/or* hematuria ≥ 5 RBC/HPF *and/or* eGFR < 90 mL/min/1.73 m^2^ [31]. Accordingly, inactive LN was defined as NIH-AI score < 4 and UPCR < 0.5 plus no hematuria (0–4 RBC/HPF) when NIH-AI scores were or were not available within 3 months of a study visit. Complete response to induction therapy, or complete kidney remission, was defined as NIH-AI as < 4 on repeat kidney biopsy. If repeat biopsy was not available, then complete response was defined as UPCR < 0.5 *plus* GFR > 90 mL/min/1.73 m^2^ *plus* inactive urine sediment (no hematuria) [31]. Partial response to induction therapy, or partial kidney remission, was defined as UPCR improvement by ≥ 50% *plus* at least stable eGFR compared to LN diagnosis. Those patients with LN who failed to achieve a partial or complete response to induction therapy were considered non-responders.

### Statistical analysis

In patient-level variables, descriptive analyses are reported and compared by Student’s *t*-test (mean ± SD) or ANOVA test (mean ± SD). The Wilcoxon test (median, IQR) or Kruskal–Wallis test (median, IQR) was performed to compare group differences for continuous variables for patients with LN (active or inactive LN), SLE patients without known LN (no-LN), and age-matched healthy controls. Matching controls were recruited to address potential sources of bias. Study size was determined by prior studies using biomarkers. Fisher’s exact or chi-square tests, as appropriate, were performed for testing of group differences of categorical variables. Correlation was established via Pearson’s correlation coefficient, with *r* of 0.1–0.39 representing weak correlation, 0.4–0.69 representing moderate correlation, 0.7–0.89 representing strong correlation, and greater than 0.9 representing very strong correlation [32]. Baseline (at the time of enrollment) demographics and SLE characteristics were compared for each clinical group (LN, no-LN, and control). Visit-level variables were compared by patient’s age and urinary biomarkers by clinical groups by general estimating equation (GEE) to account for repeated measures within patients. Paired *t*-test was performed in evaluating patient changes of pRAIL scores from the start to the end of induction therapy for LN patients classified as LN responders (complete and partial) during induction therapy, but not for non-responders. Two-sided *p*-values < 0.05 were considered statistically significant.

Prior to use in the RAIL algorithm, biomarker concentrations were natural-log transformed. The pRAIL algorithm was used only for pediatric and young adult cSLE patients up to age 23 years at enrollment, as previously published [13, 14]. The pRAIL we evaluated produced standardized and non-standardized scores [13, 14]. The standardized RAIL score refers to the score that utilized creatinine standardized urinary biomarkers, while the non-standardized score used absolute values of the urinary biomarkers. All statistics were performed using SAS (version 9.4, Cary, NC, USA).

## Results

A total of 128 pediatric or young adult patients (< 24 years of age; LN: 70; no-LN: 38, controls: 20) were included for the analyses of changes of pRAIL scores. The study duration was from June 2019 to December 2021. Among 70 lupus nephritis patients, 32 patients were undergoing induction therapy for LN, of which 25 met our criteria for active LN, while the remaining 7 did not due to NIH-AI scores < 4.

### Patient disposition and baseline characteristics of the pediatric and young adult patients

Table 1 shows the baseline characteristics of the pediatric and young adult patients included in pRAIL analyses. The median age at diagnosis of cSLE was 14 years, and most were female. Disease activity was significantly higher in the pediatric LN group as compared to the pediatric no-LN group (median total SLEDAI score; 10 vs 2, *p* < 0.0001). Most pediatric and young adult LN patients had proliferative LN with a median NIH-AI score of 7 (IQR: 2, 12) and NIH-CI score of 1 (IQR: 0, 3) on kidney biopsy, respectively.

**Table 1:** Baseline demographics and lupus characteristics of pediatric population

*n* (% of *N*) OR median (IQR)	(A) LN (*N* = 70)	(B) no-LN (*N* = 38)	(C) Controls (*N* = 20)	*p*-value^†^	
				*(A) vs. (B)*	*(A) vs. (B) vs. (C)*
Age at visit (years)	16.0 (13.0, 19.0)	16.9 (15.6, 19.11)	10 (7.5, 13.5)	0.30	< .0001
Sex -Female	59 (85.5%)	32 (88.9%)	8 (34%)	0.63	< .0001
Ethnicity—% Hispanic	19 (27.1%)	1 (2.6%)	1 (5%)	0.0004	< .0001
Race —% White	18 (25.7%)	19 (50%)	10 (50%)	0.01	0.0007
SLEDAI total score	10 (2, 22)	2 (0, 6)	–	< .0001	–
Renal SLEDAI	4 (0, 12)	0 (0, 0)			
Prednisone (mg/day)	26/15.5 (10, 40)	16/20 (10, 25.5)	–	0.97	–
Disease duration	0.1 (0, 3.12)	1.68 (0.5, 3.13)	–	< 0.05	–
ISN/RPS class, *n* (% of 70)					
Class I/II	9 (12.9%)				
Class III	18 (25.7%)				
Class IV	24 (34.3%)				
Pure class V	5 (7.1%)				
Class III/IV + V	8 (11.4%)				
Unknown	6 (8.6%)				
NIH-AI score (*n* = 41) ‡	7 (2, 12)				
NIH-CI score (*n* = 41) ‡	1 (0, 3)				

^†^By Wilcoxon rank-sum test or Kruskal–Wallis tests for continuous variables, and by chi-square test used for categorical variables. ^‡^NIH-AI and NIH-CI scores were available for only 41 of the 70 patients with LN

### Urine biomarker concentrations and RAIL score by LN status

The pRAIL scores significantly differed by LN status (active LN, inactive LN, no-LN) and by healthy controls. Table 2 summarizes individual urine biomarker concentrations contributing to the pRAIL score, with statistically significant differences with active versus inactive LN, active LN versus no-LN, and SLE (active LN, inactive LN, no-LN) versus controls. Only urine levels of NGAL, adiponectin, and KIM-1 were statistically significantly higher in the no-LN group compared to healthy controls (all *p* < 0.03). The pRAIL scores differed between the active LN group and other groups but not between the inactive LN group versus the non-LN group, irrespective of whether pRAIL scores were standardized by urine creatinine or not.

**Table 2:** Urine biomarker and pRAIL scores by LN status and in healthy controls

Variables (mean ± SD)	(A) Active LN (*N* = 149 visits)	(B) Inactive LN (*N* = 228 visits)	(C) Non-LN (*N* = 113 visits)	(D) Healthy (*N* = 20 visits)	*p*-value^#^			
					*(A) vs. (B)*	*(A) vs. (C)*	*(B) vs. (C)*	*(C) vs. (D)*
Age at visit (years)	16.77 ± 3.18	16.51 ± 3.51	17.33 ± 3.59	10.9 ± 4.3	0.4508	0.3760	0.1314	< .0001
NGAL (ng/mL)	3.34 ± 0.87	2.99 ± 0.9	3.26 ± 1.08	2.39 ± 0.34	0.0009	0.5517	0.0296	0.0014
Ceruloplasmin (ng/mL)	6.14 ± 2.4	3.57 ± 2.39	3.45 ± 1.03	3.07 ± 0.97	< .0001	< .0001	0.6207	0.2554
MCP-1 (pg/mL)	6.62 ± 1.44	5.37 ± 1.36	5.42 ± 1.66	4.03 ± 1.76	< .0001	< .0001	0.7867	0.0016
Adiponectin (ng/mL)	4.76 ± 2.24	2.69 ± 1.99	3.92 ± 1.8	2.27 ± 1	< .0001	0.0013	< .0001	0.0002
Hemopexin (ng/mL)	7.33 ± 1.29	5.9 ± 1.32	6.01 ± 0.93	5.38 ± 0.89	< .0001	< .0001	0.4213	0.0121
KIM-1 (pg/mL)	6.87 ± 1.22	6.05 ± 1.32	5.61 ± 1.54	4.28 ± 1.67	< .0001	< .0001	0.0064	0.0012
Creatinine (mg/mL)	4.56 ± 1.02	4.54 ± 1.15	4.53 ± 0.89^	3.5 ± 0.88	0.9076	0.9076	0.9076	< .0001
pRAIL score	0.46** ± **1.46	− 0.34** ± **1.56	− 0.51** ± **1.9	− 2.14** ± **2.32	< .0001	< .0001	0.4034	0.0017
Standardized pRAIL score	− 2.46** ± **1.41	− 3.25** ± **1.29	− 3.35** ± **1.51	− 4.38** ± **1.89	< .0001	< .0001	0.5205	0.0164

^#^ANOVA test or *t*-test used for continuous variables. *p*-values were adjusted for the pairwise multiple comparisons. Pediatric RAIL (with or without creatinine standardization) =  − 4.29 − 0.34 ×  − NGAL − 0.06 × ceruloplasmin + 0.89 × MCP-1 + 0.18 × adiponectin − 0.65 × hemopexin + 0.62 × KIM-1

**Fig. 1:**
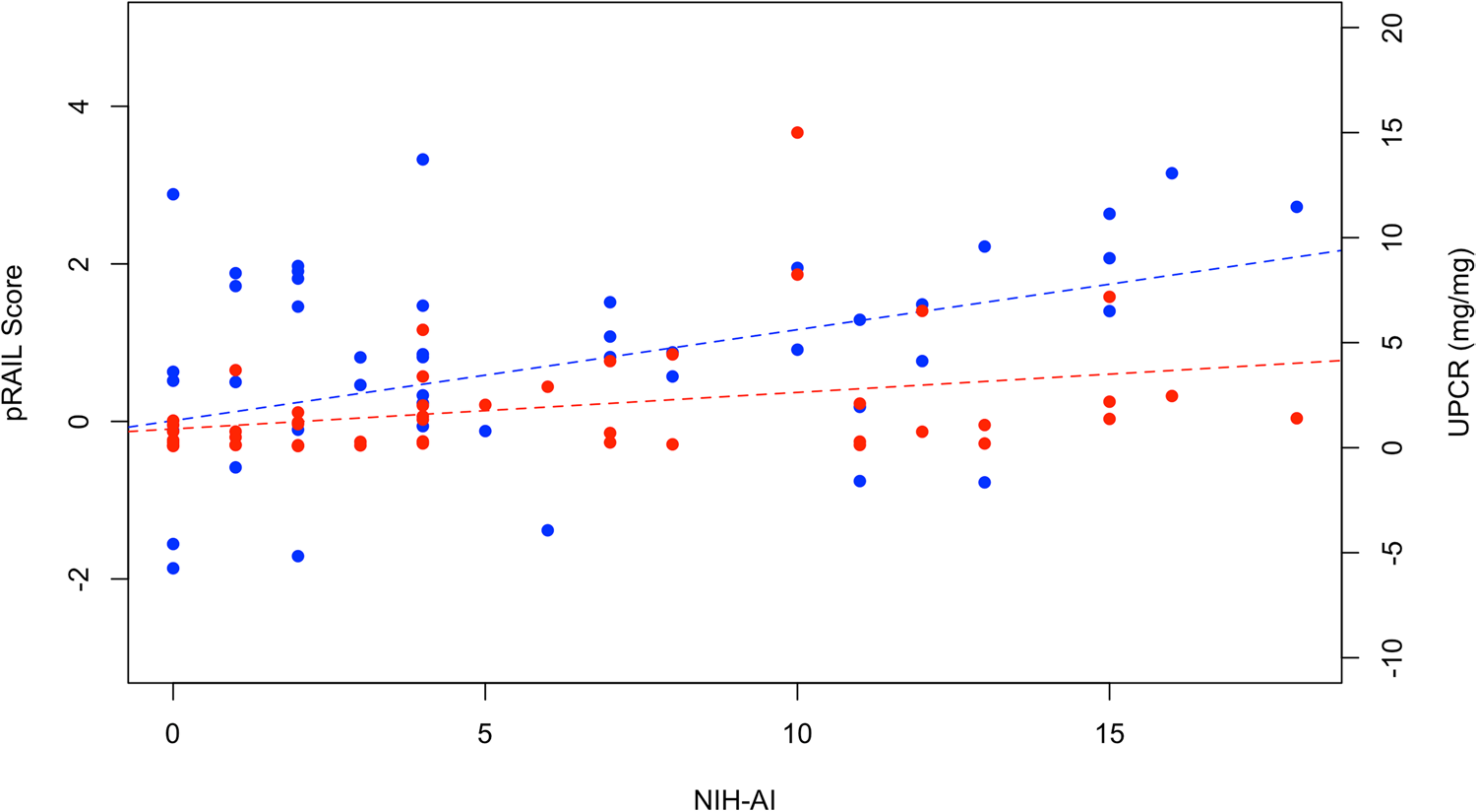
UPCR in mg/mg (orange, *r* = 0.33) and pRAIL scores (blue, *r* = 0.40) for all patients with active disease (NIH-AI) diagnosed by biopsy

There was only a weak correlation between pRAIL scores and UPCR, with an *r* value of 0.14. Considering the subset of active LN patients with available NIH-AI scores, pRAIL scores were moderately (*r* = 0.4; *p* = 0.0054) and UPCR weakly (*r* = 0.33; *p* = 0.02) correlated with NIH-AI scores as shown in Fig. 1.

### Changes in RAIL scores with induction therapy

There were 32 analyzable pediatric or young adult LN patients who enrolled at the time of a kidney biopsy, among them 7 patients whose LN was classified as “inactive” (NIH-AI score < 4) and therefore excluded, and 25 patients who had active LN at the start of induction therapy. Immunosuppressive therapy for active LN included intravenous cyclophosphamide (*n* = 14) and oral mycophenolate mofetil (*n* = 11). There were 22 responders to induction therapy, of whom 15 patients met criteria for complete response, 7 patients were partial responders, and 3 were non-responders to induction therapy. Distribution of age at baseline, gender, race, ethnicity, and ISN/RPS classes were all similar across groups. Proliferative nephritis was evenly distributed across response status. Of the 17 patients with class III or IV LN, 2 patients (66.67%) were non-responders, 5 patients (71%) were partial responders, and 10 patients (66.67%) were complete responders. Finally, baseline pRAIL scores were similar between partial and complete responders (Table 3).

**Table 3:** Characteristics and biomarkers of complete and partial responders in pediatric patients during induction therapy^‡^

	Partial responder (*N* = 7)			Complete responder (*N* = 15)		
	Baseline	End of induction therapy	*p*-value^#^	Baseline	End of induction therapy	*p*-value^#^
Age at visit (years)	16.7 ± 1.53	17.15 ± 1.54	–	14.21 ± 2.6	14.74 ± 2.65	**–**
UPCR (mg/mg)	3.32 ± 2.15	1.66 ± 1.85	0.27	4.14 ± 4.79	0.76 ± 1.66	< 0.01
eGFR (mL/min/1.73 m^2^)	104.17 ± 59.73	115.17 ± 49.23	0.47	95.18 ± 39.73	107.42 ± 17.58	0.26
*pRAIL score*	0.80 ± 0.88	0.87 ± 1.13	0.91	0.77 ± 1.71	− 0.3 ± 1.32	0.03
*Standardized pRAIL score*	− 1.91 ± 0.77	− 2.5 ± 0.9	0.15	− 2.16 ± 1.33	− 3.31 ± 1.15	0.008

^†^Values are mean ± SD; ^†^standardization by urine creatinine. ^#^Paired *t*-test; complete responder: UPCR < 0.5 and eGFR > 90 mL/min/1.73 m^2^ plus no hematuria; partial responder UPCR improved by 50% with at least stable eGFR. eGFR provided by cystatin C or calculated by modified Schwartz equation. ^‡^Visit 1: diagnosis of LN at the time of kidney biopsy; visit 2: at end of induction therapy around months 6–9

**Fig. 2:**
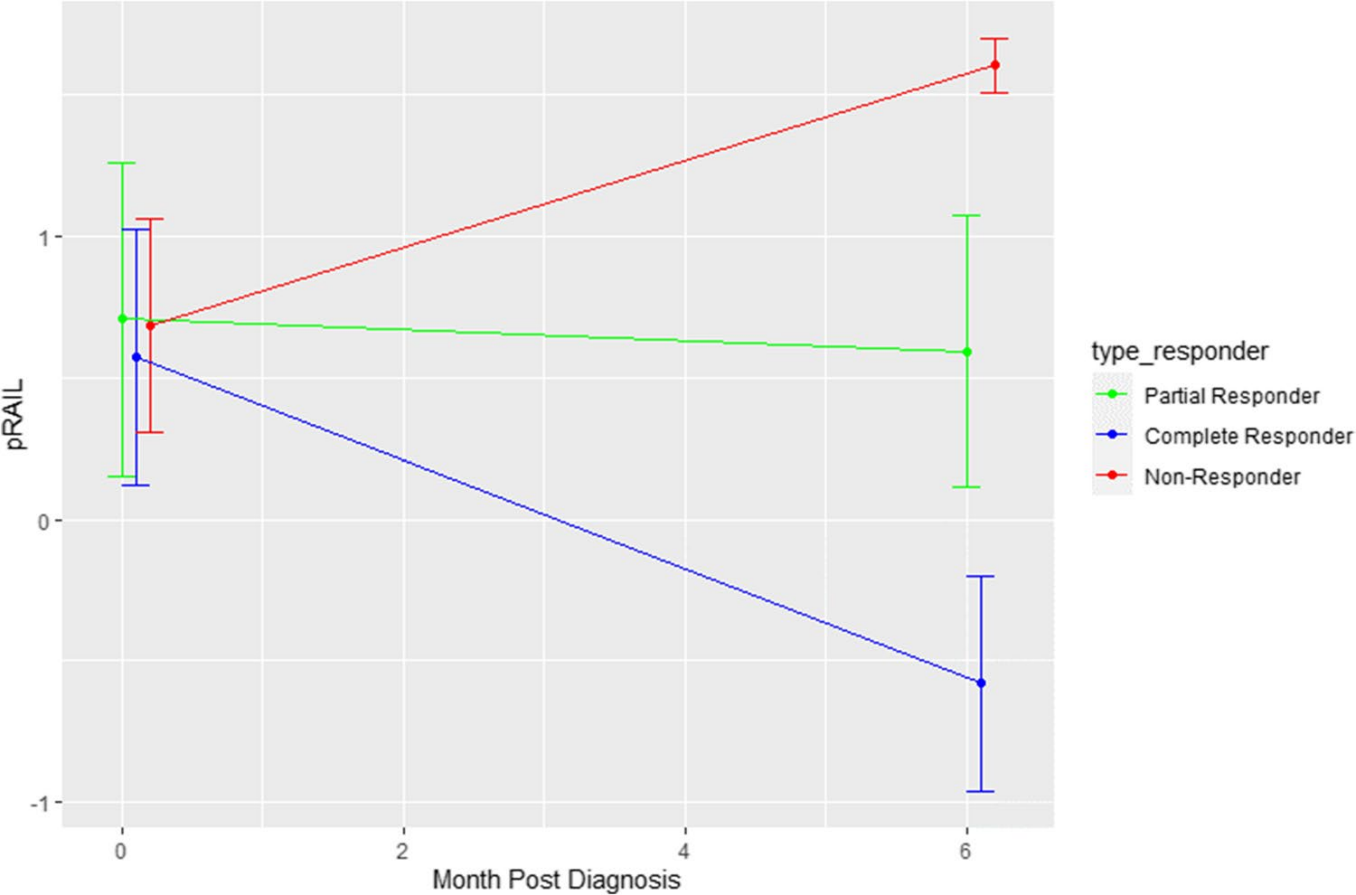
The change in absolute RAIL score by responder status. Complete responders had decrease in pRAIL scores, partial responders had no change, and non-responders had an increase in pRAIL scores

Among the three non-responders, standardized and non-standardized pRAIL scores numerically increased during induction therapy (see Fig. 2). Conversely, mean ± SD of pRAIL scores showed a statistically significant decrease in complete responders by 1.07 ± 1.7 (*p* = 0.03). For partial responders, there was no change of the pRAIL score during induction therapy (0.07 ± 1.56, *p* = 0.91).

**Fig. 3:**
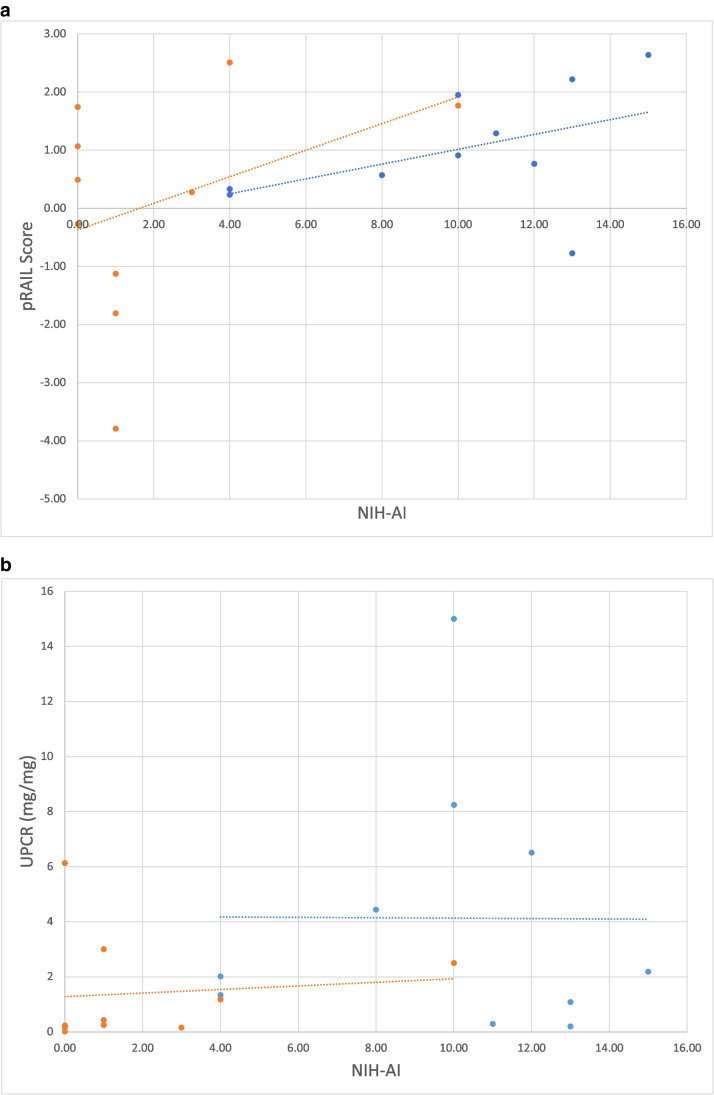
**A** NIH-AI score available during induction therapy by the pRAIL score. The blue line shows pre-treatment values (*r* = 0.46) and the orange line shows post-treatment values (*r* = 0.37). **B** Available NIH-AI by UPCR in mg/mg during induction therapy. The blue line shows pre-treatment values (*r* = 0) and the orange line shows post-treatment values (*r* = 0.1)

Ten patients underwent a repeat kidney biopsy upon completion of induction therapy, including nine with complete renal response and 1 non-responder. The mean ± SD of NIH-AI scores decreased from 9.89 ± 3.92 to 1.1 ± 1.45 among complete responders during induction therapy (*p* = 0.0007). Overall, mean ± SD absolute pRAIL scores of complete responders verified by repeat kidney biopsy decreased by 1.08 ± 1.13, and standardized pRAIL scores by 0.43 ± 0.31, respectively. The average UPCR pre-therapy was 4.56 ± 4.74 and the average UPCR post-biopsy was 1.29 ± 2.04. As shown in Fig. 3, the pRAIL score demonstrates better correlation with NIH-AI both pre- and post-treatment (*r* = 0.46 and 0.37, respectively), while UPCR had no correlation with NIH-AI at either time point, with *r* values of 0 and 0.1, respectively.

The pRAIL score, both absolute and standardized, can distinguish active, inactive, and absent lupus nephritis in the pediatric population. We now show that the change in pRAIL score detects improvement in disease activity during induction therapy, and that this pRAIL difference score corresponds with change in the NIH-AI on biopsy that may not be detected by current biochemical measures, including UPCR measurements.

## Discussion

In this study, we aimed to assess the ability of the pRAIL score to detect response to induction therapy. We confirmed that both absolute and standardized pRAIL scores differentiate patients with active LN from those with inactive LN, or SLE patients without kidney involvement. Healthy controls have significantly lower RAIL scores than SLE patients, even those without known kidney disease. Complete response of active LN to induction therapy is associated with a mean decrease of the standardized pRAIL score of at least 0.5, irrespectively of the degree of the baseline histological activity. Finally, the pRAIL score is superior to proteinuria in correlating with activity as found on biopsy.

We had previously shown that both absolute and standardized pRAIL scores were similarly well suited to discriminate moderate to highly active LN (NIH-AI > 10) from LN with lesser degrees of inflammation as seen on kidney biopsy [11, 14]. Using current standard clinical measures of LN, pRAIL scores clearly differentiate active from inactive LN [11, 14]. This study builds on this body of evidence as we delineate changes in pRAIL scores, reflective of partial and complete response to induction therapy. Given that decreases in standardized rather than absolute pRAIL scores were consistent, irrespective of baseline kidney inflammation (NIH-AI score), standardized pRAIL scores might be better suited to assess response to LN therapy. We suspect that because of the small group of partial LN responders (*n* = 7), we were unable to show statistically significant decreases in standardized pRAIL scores in this subgroup.

Another reason for the small changes in pRAIL scores in partial LN responders may be the choice of the response definition selected for this study, which was based on a prior consensus formation exercise in children with LN [33]. Nonetheless, there are no generally accepted, standard definitions for complete or partial response to induction therapy in either pediatric or adult LN [5, 34]. Choices of the response definitions influence the frequency of LN response in adult LN patients as used in clinical care and research [5, 34–39], and the same holds true for pediatric LN [5]. In this context, RAIL scores will provide additional support for the biologic relevance of the observed response of LN to a given treatment intervention. Importantly, corrected for treatment response, we have shown consistently in the past that changes in RAIL scores are not influenced by the type of concurrent therapies, including the use of proteinuria-sparing agents [12–14, 40].

Patients who achieve partial LN response have better outcomes than non-responders, but have worse outcomes than complete LN responders [38]. If confirmed in larger studies, changes in pRAIL score might be useful to escalate immunosuppressive therapy early in LN patients without robust decreases of pRAIL scores as would be expected in partial or non-responders to induction therapy.

The degree of histological activity on kidney biopsy at the time of LN diagnosis or on repeat biopsy is an established prognostic factor for the longer-term outcomes of LN [25, 26, 29, 39, 41]. Given that RAIL scores reflect the level of inflammation, measuring RAIL biomarkers may provide a non-invasive measure to intervene and provide additional therapies, with the goal to improve kidney outcomes in LN. Proteinuria, particularly following treatment of active inflammation, may reflect chronic damage to the kidney as a result of their presentation that may respond to non-immunosuppressant therapy [42, 43]. Combining the RAIL score with urine protein measurement may provide insight into the health of the kidney following induction therapy. Conversely, there is a subset of patients who may have resolution of proteinuria but have active disease on repeat biopsy and the addition of the RAIL score into practice may assist with decisions to continue immunosuppressive therapies [7].

In this study, levels of urine biomarkers and RAIL scores were lower in age-matched healthy controls compared to SLE patients, irrespective of kidney involvement. Lower levels of kidney biomarkers and RAIL scores could be considered a reflection of common, subclinical kidney inflammation in SLE patients, even those without abnormal urinalysis.

There are several limitations to this study. First, we only had access to patients with available repeat kidney biopsy at the end of induction therapy, requiring reliance on other definitions of active/inactive LN. This was an observational study, so we relied on data from real clinical encounters; thus, study results may be biased due to missing data and informational bias. There were a small number of non-responders, limiting the analyses that could be performed in the induction group. However, the observations in this study were consistent with those of our prior validation studies of the RAIL [11, 13, 14]. Additionally, all the urine biomarkers were performed on ELISAs, which are single-plex assays. These are time-consuming and more expensive and use more sample than a multiplex assay [44, 45]. We have previously shown that multiplex assay using Luminex is feasible for four of the six biomarkers (NGAL, KIM-1, MCP-1, adiponectin) with excellent correlation [46] and work is ongoing to create a multiplex assay for all 6 urinary biomarkers for use in the clinical realm.

In conclusion, we provide evidence for the value of a composite urinary biomarker score and RAIL to support the severity of LN and its response to induction therapy.

## Supplementary Information

Below is the link to the electronic supplementary material.

Graphical Abstract (PPTX 54 KB)

## Data Availability

The datasets generated during and/or analysed during the current study are available from the corresponding author on reasonable request.
