# Supplementary material for: Urine biomarker score captures response to induction therapy with lupus nephritis
Source: Pediatr Nephrol. 2023 Jan 30;38(8):2679–88. doi: 10.1007/s00467-023-05888-z (PMC10393841; doi:10.1007/s00467-023-05888-z)
Supplement: Supplementary file 1 — Graphical Abstract (PPTX 54 KB) [file 467_2023_5888_MOESM1_ESM.pptx]

## Slide 1
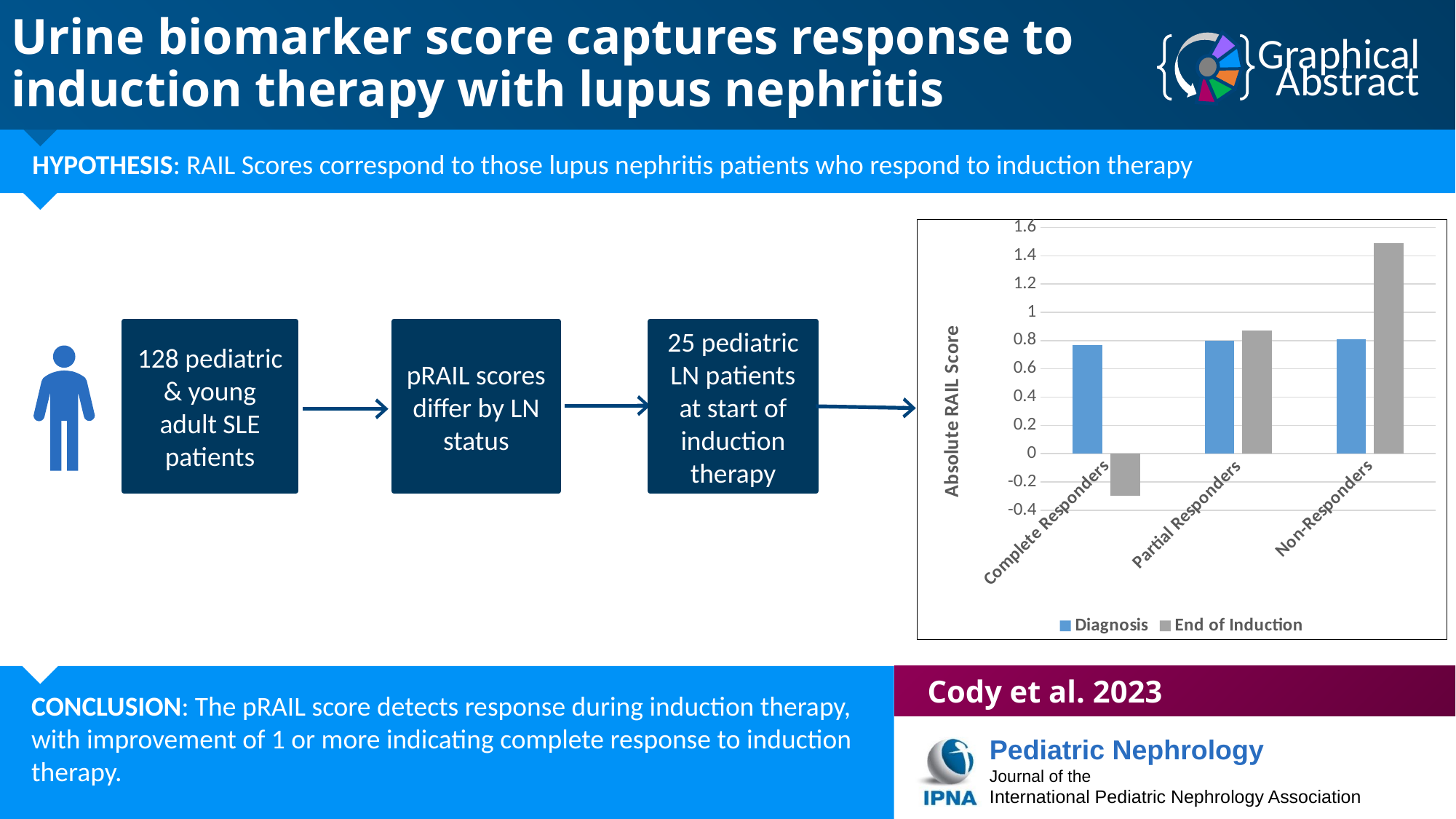

Urine biomarker score captures response to induction therapy with lupus nephritis
HYPOTHESIS: RAIL Scores correspond to those lupus nephritis patients who respond to induction therapy
### Chart
| Category | Diagnosis | End of Induction |
|---|---|---|
| Complete Responders | 0.77 | -0.3 |
| Partial Responders | 0.8 | 0.87 |
| Non-Responders | 0.81 | 1.49 |128 pediatric & young adult SLE patients
pRAIL scores differ by LN status
25 pediatric LN patients at start of induction therapy
Cody et al. 2023
CONCLUSION: The pRAIL score detects response during induction therapy, with improvement of 1 or more indicating complete response to induction therapy.
